# An update on neuro-otology and neuro-ophthalmology – focusing on new developments in the last three years

**DOI:** 10.1007/s00415-026-14153-w

**Published:** 2026-10-11

**Authors:** Alexander A. Tarnutzer, Michael L. Strupp, Louisa Lehner

**Affiliations:** 1 Faculty of Medicine, University of Zurich, Zurich, Switzerland; 2 Cantonal Hospital Baden, 5404 Baden, Switzerland; 3 Department of Neurology, LMU University Hospital, LMU Medizin, LMU, Munich, Germany

**Keywords:** Vestibular, Stroke, Vertigo, Dizziness, New therapies, Recent developments, Differential diagnosis

## Abstract

Vertigo, dizziness, and imbalance are among the most frequent presenting symptoms in primary care and in the emergency department (ED). The clinical diagnosis is based on a structured patient history and bedside examination of the vestibular system and the ocular motor system, looking especially for central ocular motor signs. Recent guidelines, such as the GRACE−3 guideline on acute vertigo and dizziness, the increasing availability of bedside diagnostic algorithms, and the use of quantitative vestibular and ocular motor testing in both the ED-setting and in the outpatient center have facilitated and standardized the approach to the dizzy patient. Here we review recent developments in neuro-otology and neuro-ophthalmology, covering the period after the latest neuro-otology update was published in this journal in 2023. Both new-onset acute, episodic and chronic vestibular conditions are discussed separately, highlighting advancements in disease characterization, diagnostic criteria, and treatment strategies. While major progress has been made in terms of the pathophysiology, etiology, genetics, and diagnostic criteria of peripheral, central, and functional vestibular diseases as well as ocular motor disorders, this knowledge has to be transferred into clinical practice. Systematic teaching of doctors to increase knowledge and to improve skills in applying the different tests in clinical practice on a high level are identified challenges. In terms of treatment, there is still a very great need for state-of-the art clinical trials applying approved therapies.

## Introduction

Vertigo, dizziness, and imbalance are amongst the most frequent presenting symptoms both in primary care and in the emergency department (ED). The clinical diagnosis is based on a structured patient history (including time course, triggers/modulating factors, and accompanying symptoms) and bedside examination of the vestibular system (nystagmus, head-impulse test, diagnostic positional maneuvers, and Romberg test) and the ocular motor system, looking for central ocular motor signs, including the cross cover tests. The diagnostic criteria, elaborated by the International Classification Committee (ICVD) of the Bárány-Society, highlight these aspects and have turned out to be very helpful in clinical practice. In addition, recent guidelines, such as the guidelines for reasonable and appropriate care in the emergency department 3 (GRACE−3) on acute vertigo and dizziness [1] and guidance papers for both ED and primary care [2] provide valuable tools. At the same time, the increasing availability of bedside diagnostic algorithms and the use of quantitative vestibular and ocular motor testing in both the ED-setting and the outpatient center allow a more standardized approach. This facilitates the detection of more subtle findings and enables access to experts by telemedicine, helping to close some of the management gaps. Here we review recent developments in neuro-otology and neuro-ophthalmology, covering the period after the latest neuro-otology update was published in this journal in 2023 [3].

This update is structured based on the clinical presentation of patients: first, acute onset of symptoms; second, episodic symptoms; and third, persisting symptoms.

### Acute onset of vertigo, dizziness and gait imbalance

#### Epidemiology

Amongst all ED consultations related to vertigo or dizziness, about 10% of patients meet diagnostic criteria for an acute vestibular syndrome (AVS, defined as a “clinical syndrome of acute-onset, continuous vertigo, dizziness, or unsteadiness lasting days to weeks and generally including features suggestive of new, ongoing vestibular system dysfunction. such as vomiting, nystagmus, severe postural instability" [4]). Another presentation is acute imbalance syndrome (AIS), defined as an AVS without nystagmus [5]. About one fifth of those patients will eventually receive a diagnosis of stroke according to a systematic review [6]. Recent developments characterizing the spectrum of AVS/AIS cases and how to distinguish peripheral-vestibular from central-vestibular cases are reviewed here. In another systematic review, 49.4% of central AVS cases were classified as AIS [5]. Whereas AIS presentation was rare in strokes affecting the territory of the anterior inferior cerebellar artery (AICA, 9%), fractions reached 40% (posterior inferior cerebellar artery (PICA)), 57% (superior cerebellar artery (SCA)), and 62% (posterior cerebral artery (PCA)) for other vascular territories. Data on underlying causes in non-central AIS is limited, with dysautonomia, drug intoxication, and heart disease being most frequently identified. Thus, patients presenting with AIS have a distinct differential diagnosis compared to those with AVS with nystagmus, mostly due to the distribution of non-central causes.

In a recent single-center prospective study focusing on MRI-proven stroke-related acute vertigo/dizziness or imbalance, 58.7% of patients were diagnosed with AIS, whereas 38.7% received a diagnosis of AVS with nystagmus, and 2.7% of patients were classified as having central positional vertigo [7]. Interestingly, lesion-symptom mapping demonstrated distinct distributions. Specifically, AIS lesions were located in the anterior cerebellar lobe, disturbing cerebellar pontomesencephalic networks for posture and gait control. AVS lesions affected the posterior cerebellar/flocculonodular lobe and associated vestibulo-ocular reflex networks. These findings emphasize the importance of selecting bedside testing appropriately, focusing on algorithms such as STANDING, when no nystagmus is seen.

#### Acute unilateral vestibulopathy/vestibular neuritis

The current diagnostic criteria for acute unilateral vestibulopathy (AUVP) are summarized in Table 1 [8].

**Table 1:** Diagnostic criteria for acute unilateral vestibulopathy/vestibular neuritis [8]

Diagnostic criteria for acute unilateral vestibulopathy/vestibular neuritis [8]
A) Acute or subacute onset of sustained spinning or non-spinning vertigo (i.e., an acute vestibular syndrome) of moderate to severe intensity with symptoms > 24 h
B) Spontaneous peripheral vestibular nystagmus which is direction-fixed and enhanced by removal of visual fixation with a trajectory appropriate to the semicircular canal afferents involved (generally horizontal-torsional)
C) Unambiguous evidence of reduced VOR function on the side opposite the direction of the fast phase of the spontaneous nystagmus
D) No evidence for acute central neurological symptoms or acute audiological symptoms, such as hearing loss or tinnitus
E) No acute central neurological signs, namely central ocular motor or central vestibular signs, no pronounced skew deviation or gaze-evoked nystagmus, no acute audiological signs
F) Not better accounted for by another disease or disorder

*VOR* vestibulo-ocular reflex

According to a study in Japan, the annual incidence of AUVP is about 7.1/100,000 and has thus obviously doubled compared to earlier data from 1993, which may also be due to the better diagnosis of the main symptom dizziness [9]. About 20% of patients with AUVP already have prodromal dizziness a few hours/days before the onset of longer-lasting symptoms (study on 88 patients) [10]. Patients with a lesion of all parts of the vestibular nerve, i.e., including the inferior vestibular nerve, have more intense and longer-lasting symptoms [11] with an obviously higher risk of secondary functional dizziness (FD). Obtaining 4-h delayed post-contrast 3D-FLAIR MR-images, robust contrast-enhancement was seen in 26 of 53 patients (49%) with AUVP with the following frequency: vestibule (*n* = 20), anterior (*n* = 12), horizontal (*n* = 8), posterior (*n* = 5) semicircular canal as well as superior (*n* = 3) and inferior (*n* = 1) vestibular nerve [12]. The most relevant differential diagnosis is an acute central vestibular syndrome due to a brainstem or cerebellar infarction.

Quantitative testing of the vestibulo-ocular reflex (VOR) (in the high frequency range) by means of video head-impulse test (HIT) plays a very important role in differentiation. This is supported, for example, by a systematic review [13] and is recommended as the first instrumental vestibular test in AVS patients. A VOR < 0.7 is recommended as a "cut-off value" for the affected side and to differentiate from a central lesion, which is in accordance with the above-mentioned diagnostic criteria.

The therapy of AUVP is based on three principles:

1. Symptomatic treatment, e.g., with dimenhydrinate (for a maximum of 1–3 days).
2. "Causal therapy" with steroids; this is also recommended in the guideline of the "American Society for Academic Emergency Medicine" [1]. A recent placebo-controlled study showed no effect on the recovery of peripheral vestibular function [14], but in total only a quarter of the steroid dose was given compared to another randomized controlled trial (RCT) from 2004 [15]. So, it remains a caveat: further placebo-controlled studies with sufficient dosages and valid clinical endpoints are necessary.
3. Physiotherapy: In addition to the recovery of peripheral vestibular function, multimodal central compensation, which can be promoted by physiotherapy and possibly by medication (although the RCTs on the latter are still unconvincing [16, 17]), is an important factor in improving the symptoms of patients with AUVP. The current guideline of the "Academy of the American Physical Therapy Association" recommends the performance of balance exercises for AUVP, which can be learned with guidance and carried out independently at home [18]. Effective therapy can be achieved—with moderate to weak evidence—at a frequency of one treatment per week with instructions for independent practice three times a day over a total duration of at least twelve minutes. In this context, the training of rapid horizontal and vertical head movements, which lead to an increase in vestibular tone imbalance (the driving force for central compensation), is particularly relevant. A recent RCT shows that an individual training program, including head rotations, three times a day for eight weeks is probably the most effective therapy [19]. Importantly, there is no evidence for the effectiveness of isolated eye movements (such as slow gaze sequences or saccades) performed in a static head position in peripheral vestibular diseases. An animal study to improve central compensation showed a significant dose-dependent effect of betahistine with significant enhancement of effect by combination with selegiline. Micro-FDG-PET was also used to identify the areas in the brainstem and cerebellum that are relevant for the pharmacological improvement of the central combination [20]. Randomized trials are indicated on the basis of these pre-clinical findings.

### Acute central-vestibular causes—predictors for central structural (ischemic) AVS

In the clinical assessment of the dizzy patient, focusing on those signs that improve diagnostic accuracy and reliably detect central causes is key. Recent studies assessed the diagnostic value of spontaneous nystagmus, new-onset hearing loss, bedside diagnostic algorithms, and quantitative (video-oculography [VOG] based) testing.

#### The predictive value of spontaneous nystagmus (SN) in acutely dizzy patients

With regards to SN-patterns in AVS patients at the bedside, (isolated) vertical and/or torsional SN is found in only a minority (15.1%). However, when present, it is highly predictive for a central cause (specificity = 97.7% [95% confidence interval (CI) = 95.1–100.0%]) [21]. In contrast, a horizontal-torsional SN does not allow for a distinction between peripheral and central causes and a combined torsional downwardly beating pattern may also be observed in cases with isolated lesions of the inferior branch of the vestibular nerve.

#### Combined acute audio-vestibular loss

AVS may present in combination with new-onset unilateral hearing loss, which has been considered a central sign as reflected in the HINTS+ (head impulse, nystagmus, test-of-skew, new-onset unilateral hearing loss) algorithm [22]. Diagnosing combined acute audio-vestibular loss remains a challenge. While a combined audio-vestibular deficit makes an inner ear pathology most likely, this does not necessarily exclude a vascular pathomechanism that may be a harbinger of future sinister events. This is especially true for strokes within the territory of the AICA, because the labyrinth receives its vascular supply most often via its branches [23]. Isolated labyrinthine stroke, however, will remain undetected on standard brain MRI-DWI (diffusion-weighted imaging) sequences. Recent attempts to improve the diagnostic accuracy in these patients were mixed. In one case series of AVS patients, acute unilateral hearing loss was found in 12.7% using audiometry. Notably, the hearing loss was not noticed by the patient in 55% of cases and the presence of hearing loss did not improve the sensitivity of the HINTS to detect central (ischemic) causes in this study [24]. In contrast, in a study focusing on AVS patients with hearing loss and multiple vascular risk factors, a bilaterally positive video head-impulse test (vHIT) was supportive of an AICA infarction [25].

#### How to address acutely dizzy patients presenting without nystagmus?

While in the initial studies on the diagnostic accuracy of HINTS in AVS [26] the presence of either SN or gaze-evoked nystagmus was mandatory, a more recent definition of AVS makes nystagmus a non-mandatory feature [4]. Both for infratentorial and supratentorial pathologies leading to acute vertigo or dizziness, absence of nystagmus may pose a diagnostic challenge in the ED.

Following up on early work that has pointed to the possibility of anterior circulation stroke presenting with an AVS (even with nystagmus), this has been studied more systematically recently. In two case series, supratentorial strokes were reported in AVS patients, with absence of nystagmus in 100 [27] and 90% [28] of cases, respectively. With a fraction of 3.7% (48/1301) of all supratentorial strokes that presented as AVS in one case series, this remains an infrequent finding, which is even more true for those patients presenting with isolated vestibular symptoms (13/48, 27%) [28]. Lesion locations in supratentorial strokes were not linked to any specific (cortical) areas, but were widespread. Applying HINTS(+) in those cases poses the risk of reducing specificity for central causes.

Notably, patients presenting with isolated vertigo and acute truncal ataxia of central (mostly ischemic and infratentorial) origin, but no nystagmus may represent up to 15% of all AVS cases [29]. Likewise, between 22% and up to 50% of stroke patients presenting with an AVS may have no nystagmus [5, 29, 30]. Amongst patients with AVS linked to anterior circulation stroke, an even smaller proportion (35%) of cases demonstrated nystagmus in one case series [30]. This poses a further challenge in selecting the appropriate diagnostic algorithm in AVS patients without nystagmus. In a case series of 80 patients with confirmed posterior circulation stroke presenting as AVS, any nystagmus (spontaneous, gaze-evoked) was detected in 59% (57/80) of patients only at admission by use of VOG [31]. On follow-up, 12/33 patients that initially demonstrated no nystagmus developed a peripheral- or central-type nystagmus as early as on day two, emphasizing the additional value of follow-up VOG in those AVS patients without nystagmus on first assessment.

#### Bedside diagnostic algorithms to address the acutely dizzy patient

Fortunately, in recent years additional studies have improved our understanding of the usefulness of various diagnostic algorithms in the evaluation of patients with acute dizziness. This includes both the impact of adding new-onset unilateral hearing loss to the HINTS testing battery (termed “HINTS+”, first introduced in 2013 [22]), the use of alternative algorithms that can be applied to a broader patient cohort, such as the STANDING algorithm (first introduced in 2014 [32]), the Sudbury Vertigo Risk Score [33], or the graded rating of truncal instability (GTI) [34], and the combination of several algorithms [35].

One of the key barriers to implementing bedside algorithms in the ED is a lack of training on how to apply the single steps and how to interpret findings observed. In a recent systematic review and meta-analysis on the impact of training on the diagnostic accuracy of HINTS(+), it was demonstrated that moderately trained non-specialists were able to apply HINTS with a similarly high sensitivity compared to neuro-otology subspecialists (95.0% [91.2–98.9] vs. 94.3% [88.2–100.0]), albeit specificity was lower (89.1% [83.0–95.2] vs 97.6% [94.9–100.0]) [36]. With several studies having demonstrated successful implementation of HINTS/STANDING with as little as six hours of structured training [37, 38], this supports its applicability to the ED setting.

Due to the overlap of vestibular and auditory symptoms in both labyrinthine disease and central disorders, bedside testing should address both aspects. This is implemented in the HINTS + algorithm. In a recent systematic review, testing for new-onset unilateral hearing loss added to HINTS increased stroke-sensitivity. Importantly, this effect was mainly related to AICA-strokes with abnormal HIT, where adding acute unilateral hearing loss yielded an absolute increase of 17.8% in stroke-sensitivity [39]. Thus, in AVS, hearing should be assessed at the bedside (and any bedside hearing loss detected ideally confirmed by audiometry) in any patient with peripheral HINTS.

The STANDING algorithm is a four-step bedside procedure that requires (1) identifying SN with Frenzel/M-glasses or, alternatively, a positional nystagmus; (2) characterizing the nystagmus beating-direction in both primary and eccentric gaze; (3) assessing the VOR by the use of HIT; and (4) assessing gait [32]. Two recent studies have provided further validation of its clinical applicability in the ED. After a single training session interns could identify worrisome central causes with high diagnostic accuracy (sensitivity 84.8% [75.6%–93.9%], specificity 89.8% [82.1%–97.5%]) [37], similar as previously reported for HINTS by the same authors. Likewise, in a recent multicenter Italian study with a focus on isolated acute vertigo (i.e., without any obvious focal neurological signs), the diagnostic accuracy of the STANDING algorithm (when applied by trained emergency physicians) was substantially higher than for standard care, mainly because of a significantly lower specificity in the latter group (91.6% vs. 36.5%) [38]. Use of the STANDING algorithm also resulted in a reduced number of patients receiving a non-contrast head-CT in this study compared to standard care (48.3% vs 66.8%).

In the recently proposed “Sudbury vertigo risk score” [33], a 7-item prediction model was developed to identify those ED-patients with acute dizziness, vertigo, or imbalance that suffer from dangerous conditions, such as stroke or brain tumors. In a multicenter historical cohort validation study including 4559 patients, the sensitivity for a serious diagnosis was 100% [96.5%–100%] and the specificity was 69.2% [67.8%–70.51%] for a score of < 5. Noteworthy, the Sudbury Score has not been prospectively validated and the assessment of eye movements in this score is restricted to positional nystagmus testing for benign paroxysmal positional vertigo (BPPV). Furthermore, findings were not compared to a gold standard, such as delayed MRI-DWI or VOG.

With a focus on gait and stance, the graded truncal instability rating (GTI) allows for a bedside risk stratification (grade 0–3) of acutely dizzy patients that is part of the routine clinical assessment by emergency physicians. Thereby a distinction is made between acutely dizzy patients with an inability to stand or sit independently (grade 3), severe imbalance with standing and inability to walk without support (grade 2), mild to moderate imbalance, but ability to walk independently (grade 1), and normal walking (grade 0). In a recent systematic review, a grade 2/3 GTI had moderate sensitivity (70.8% [59.3–82.3%]) and specificity (82.7% [71.6–93.8%]) for predicting a central cause, whereas grade 3 GTI had a lower sensitivity (44.0% [34.3–53.7%] and higher specificity (99.1% [98.0–100.0%]) [34]. While the diagnostic accuracy of the GTI rating is clearly inferior to that of HINTS(+) or STANDING, combining GTI grade 2/3 with central nystagmus patterns, sensitivity was increased to 76.4% [71.3–81.6%] and specificity to 90.3% [84.3–96.3%]. Thus, the GTI rating may provide a valuable addition in settings where expertise on applying/interpreting HINTS(+) is lacking. Importantly, the combination of severe truncal ataxia and ocular lateral deviation after brief (i.e., 3–5 s duration) eye closure makes a lateral medullary (ischemic) lesion most likely [40].

**Fig. 1:**
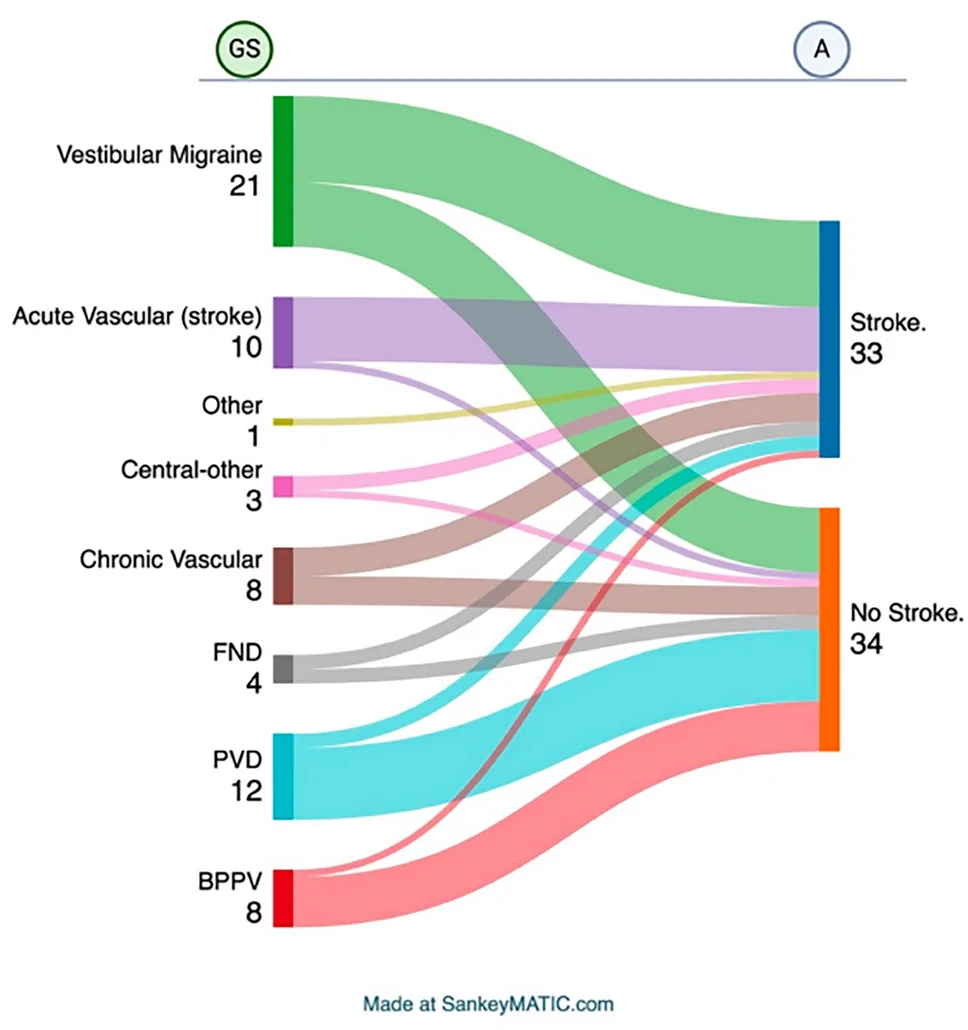
Algorithm diagnosis (A, right column) versus the clinical “Gold Standard” (GS) distribution (left column). *BPPV* benign paroxysmal positional vertigo, *FND* functional neurological disorder, *PVD* unilateral peripheral vestibulopathy. Published by [35], reused under the Creative Commons Attribution 4.0 International License

Combining targeted bedside history taking and elements of the STANDING algorithm and HINTS+ in a single algorithm has been developed for an ongoing Canadian clinical trial (NCT05176015) and was recently validated in two EDs in the UK comparing the diagnostic accuracy of trained emergency physicians and neuro-otology subspecialists [35]. The combined algorithm demonstrated a high sensitivity of 90%, whereas specificity was low (57.9%), resulting in a high rate of false positives (24/67) and a global accuracy of 62.7%. Overall, specificity was impacted by frequent misclassifications; this was especially true for conditions, such as vestibular migraine (VM) and orthostatic hypotension (see Fig. 1, from their publication [35]). While this trial is promising for further streamlining of decision-making, keeping the performance of combined algorithms as high as their single components remains a challenge. Furthermore, the effectiveness of this adapted and combined algorithm in untrained frontline providers remains unknown.

Independently of the algorithm(s) applied, re-examining patients within 24 h or even later in search of new findings and short-term resolution of initial findings is recommended, as the dynamics of clinical presentation may be of diagnostic importance.

#### Quantitative VOG-based assessments and radiographic biomarkers in acutely dizzy patients to identify central causes

**Fig. 2:**
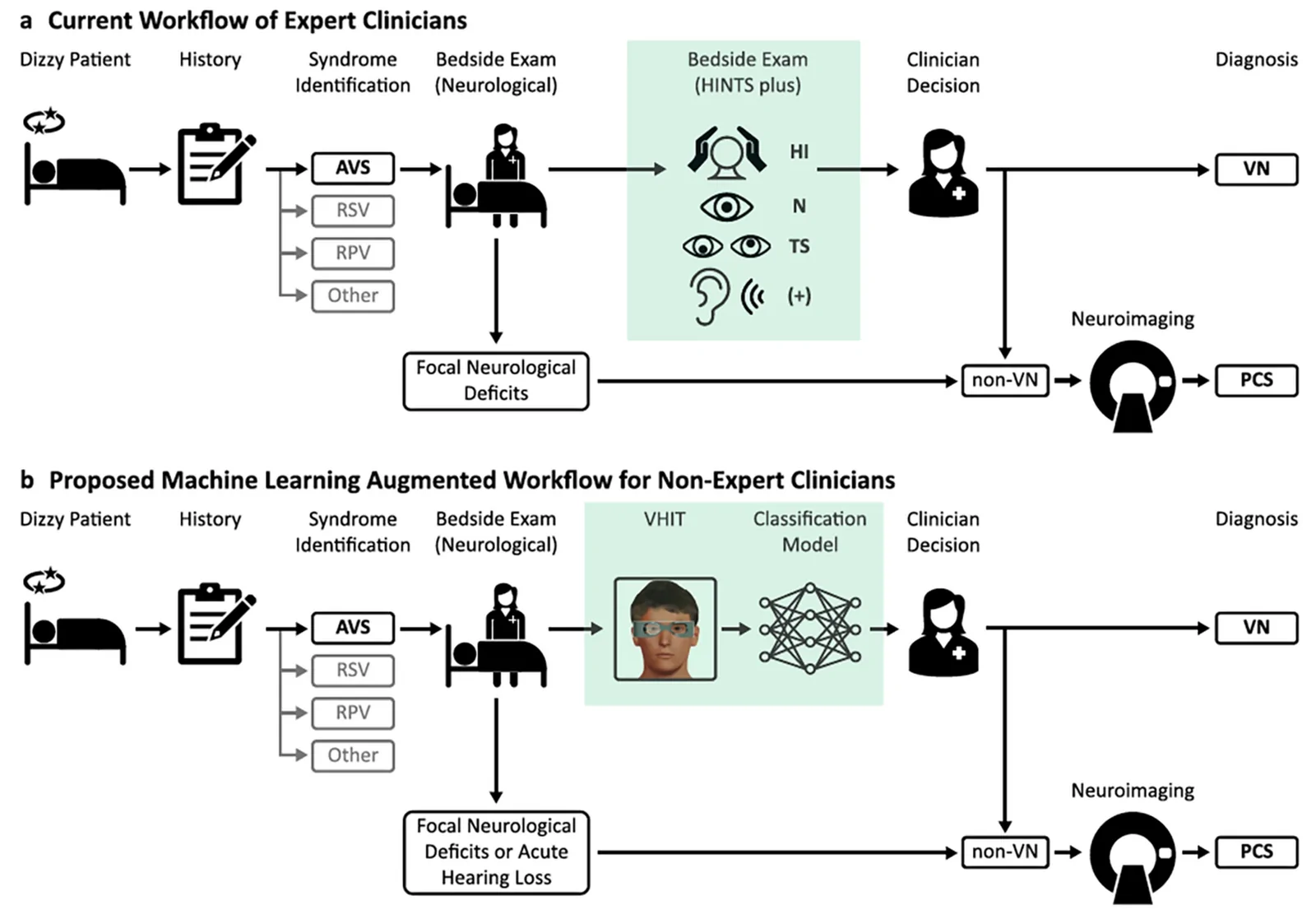
Diagnostic workflow for acute vestibular syndrome. **A** Current workflow used by expert clinicians. **B** Proposed machine learning augmented workflow for non-expert clinicians. The history separates patients with AVS from other vertigo/dizziness syndromes. Currently, expert clinicians can diagnose VN using HINTS +; non-VN cases receive neuroimaging and are treated as PCS. For non-expert clinicians who are not confident with HINTS, the authors proposed that vHIT with interpretation by a machine learning model would similarly allow accurate diagnosis. Note that the vHIT replaces HINTS and still allows for rapid clinical assessment. *AVS* acute vestibular syndrome, *HINTS* Head Impulse, Nystagmus, Test of Skew, *PCS* posterior circulation stroke, *RPV* recurrent positional vertigo, *RSV* recurrent spontaneous vertigo, *vHIT* video head impulse test, *VN* vestibular neuritis. Published by [41], reused under the Creative Commons Attribution 4.0 International License

Implementing quantitative eye movement measurements and artificial intelligence (AI) in the ED offers increased diagnostic accuracy over bedside testing in the acutely dizzy, detection of more subtle changes, and allows for telemedicine consultations as well. Using only video head-impulse test (vHIT) data, combining machine learning models and quantitative (video-based) HIT of all six semicircular canals was as accurate in distinguishing stroke from AUVP as blinded expert clinicians (accuracy of 87.8% [77.6%–95.9%] vs. 85.7% [75.5%–93.9%]) and superior to that of the optimal vHIT-gain cutoff value (75.5% accuracy [63.8%–85.7%]) (see Fig. 2 for detail, from their publication) [41]. Thus, with comparably as high diagnostic accuracy as expert clinicians, the use of vHIT-data is especially promising in more remote places without direct access to neuro-otology experts.

In a meta-analysis including horizontal-canal vHIT-data from 13 studies and 905 participants, an overall sensitivity and specificity of vHIT for detecting strokes in acutely dizzy patients of 85% [61%–95%] and 84% [70%–92%], respectively, was reported [42]. Special emphasis was put on the negative likelihood ratio of 0.18 [95% confidence interval: 0.10–0.35] and its usefulness for ruling out strokes. Potential limitations of this meta-analysis are the lack of individual patient vHIT raw-data access, the focus on vHIT-gain analysis, and varying horizontal-canal VOR-gain threshold values in included studies.

While the additional value of vHIT in the ED is well studied and evidence is solid, much less data has been published on the value of other eye movement domains in AVS patients. In a recent retrospective monocentric study, a detection model that included the gain of upward saccades, gain asymmetries of pursuit eye movements, patient age, and hypertension measures could differentiate posterior-circulation stroke from AUVP with a sensitivity and specificity of 82.8% or more [43]. However, being retrospective in nature, further prospectively collected data is needed for validation of this detection model and its impact in the diagnostic workup of acutely dizzy patients in the ED.

Horizontal radiographic conjugate gaze deviation (h-rCGD) as seen on CT- or MR-based axial imaging may arise from different mechanisms including lesions involving the frontal eye fields, brainstem/cerebellar gaze-holding networks or the vestibular nerve. While h-rCGD is a well-recognized finding in anterior-circulation stroke with large-vessel occlusion, it may also be seen in patients with AVS [44]. Importantly, unlike the presence of ocular lateral deviation h-rCGD in AVS does not allow to differentiate between peripheral and central causes. Thus, it cannot replace the bedside clinical examination including assessing for truncal ataxia and (subtle) ocular motor abnormalities in the acutely dizzy patient.

## Episodic vertigo/dizziness

### Peripheral paroxysmal positional vertigo: PPPV

An international group of authors has proposed an adaptation of the term from "benign paroxysmal positional vertigo (BPPV)" to "peripheral paroxysmal positional vertigo (PPPV)” [45]. One reason is that this most common peripheral vestibular disease is not benign in many cases: e.g., a long time to diagnosis, atypical symptoms or no symptoms at all, especially in older patients or post-traumatic PPPV („vestibular agnosia “) [46], which further extend the time to diagnosis [47], high risk of falls and recurrences and the fear of recurrence [48].

The main symptom of PPPV (please do not confuse it with persistent postural-perceptual dizziness [PPPD]), the most common cause of peripheral vestibular vertigo, is recurrent short episodes of vertigo triggered by changes in head position relative to gravity with simultaneous positional nystagmus (criteria under http://www.jvr-web.org/ICVD.html).

11% of patients with PPPV also suffer from other vestibular diseases, as a recent review (3407 patients) shows [49]: especially sudden hearing loss (12%) or AUVP (6,5%). On the other hand, many patients with other vestibular diseases additionally had PPPV: 10% of patients with AUVP and 9% of patients with Menière’s disease (MD).

PPPV most often originates from the posterior semicircular canal, however, it also originates much more frequently that thought (see below) from the horizontal canal (hc-PPPV) and very rarely from the anterior/superior semicircular canal (ac-PPPV); there are anatomical reasons for this different distribution, as the posterior semicircular canal is the relatively lowest and the superior semicircular canal is the relatively highest.

If you start testing the horizontal channels with the Supine Roll Test (SRT) with the head raised by 25°—as recommended today—the proportion of patients with an hc-PPPV is significantly higher. In a recent study in 308 patients, the proportion of the different forms was as follows: approximately 60% pc-PPPV, 24% hc-PPPV geoptropic, i.e., canalolithiasis, 14% hc-PPPV ageotropic, i.e., cupulolithiasis, and 1% ac-PPPV [50]. It is important to turn not only the head, but the whole body to the right and left, otherwise the rotation angle is too small and thus many will be overlooked, according to a study in 63% [51].

Importantly, sometimes several semicircular canals are affected, especially after a traumatic brain injury with a relatively frequent bilateral PPPV [52], so that all semicircular canals should always be examined (see below for diagnostic maneuvers). The use of smartphones to record eye movements is also very helpful (e.g., with the "EyeStabilizer App™"), as patients can perform the diagnostic maneuvers when they are symptomatic, thus improving diagnostic confidence.

Unfortunately, however, clinical practice shows that most patients with vertigo do not receive diagnostic maneuvers at all and even if PPPV is diagnosed, it is not adequately treated. Therefore, the clear recommendation is: all patients, especially older patients, with dizziness, gait disorders and falls, should be examined for PPPV and treated consistently with follow-up visits to check the effect of the therapy and the question of whether there has been a transition to another semicircular canal [53].

Up to 70% of all patients with PPPV (study with 201 patients) have "residual symptoms" after successful therapy [54]; these depend mainly on the time span between the onset of symptoms and diagnosis, as a study of 193 patients has shown [55]. This is another reason why early diagnosis is necessary.

### Therapy

Posterior canal PPPV. In the Semont maneuver (correct spelling without an acute accent), a correct rotation of the head by 45° to the frontal plane to bring the affected semicircular canal into the positional plane is important. Using an in-vitro model of a semicircular canal, it was shown that the maneuvers are only effective at an angle of 45° ± 20°. Furthermore, the further away you are from 45°, the longer the position must be maintained. Both findings are important to know for daily practice.

**Fig. 3:**
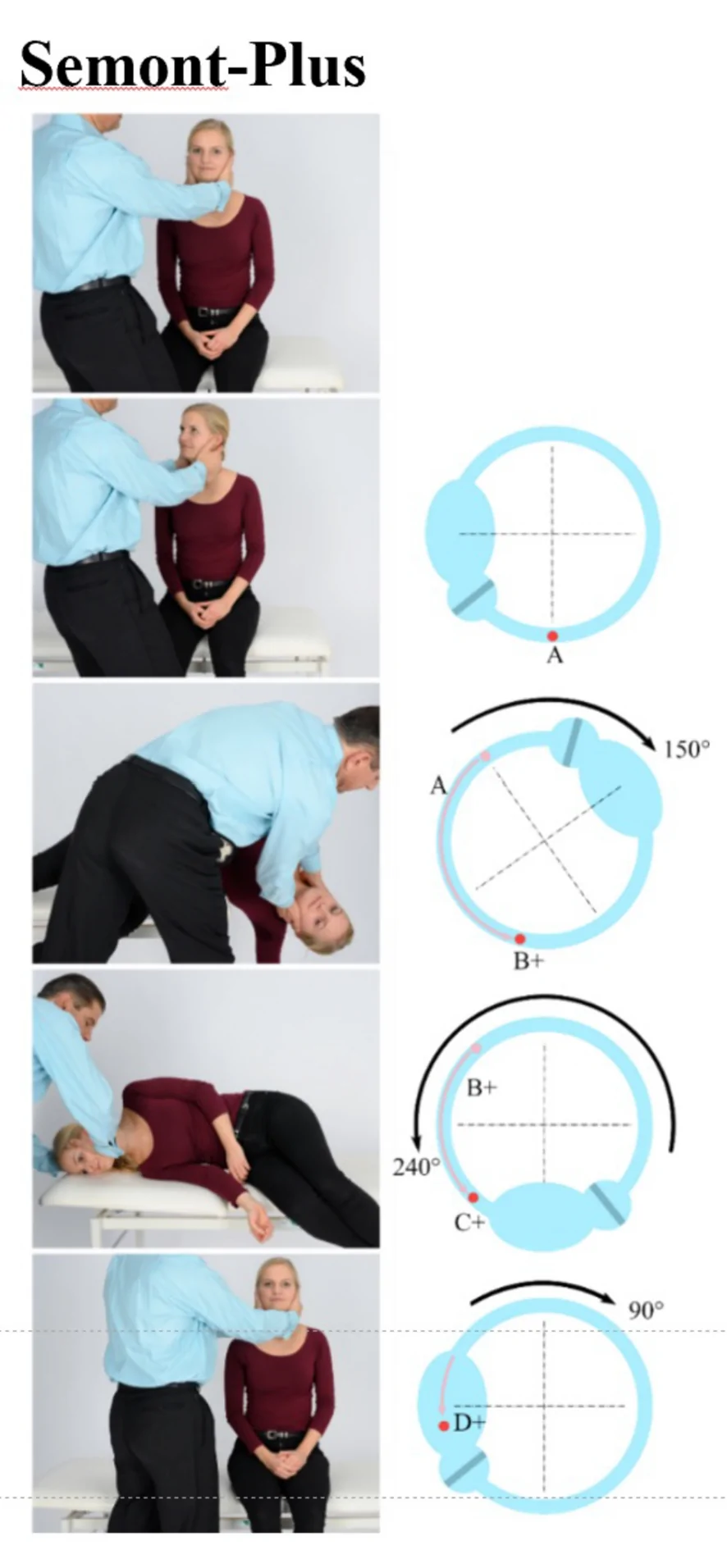
Semont-plus maneuver. In this modification, the head of the patient is moved at least 60° below earth horizontal. Based on a biophysical model and two randomized trials, this significantly increased the efficacy of the maneuver in comparison with the normal Semont maneuver and the Epley maneuver. Each position should be maintained for 1 min. The maneuvers should be repeated three times in the morning, three times in the afternoon, and three times at night until the patient cannot induce spinning vertigo on three consecutive days. On the right side, it is shown that the crystals already move beyond the vertex of the canal which is the explanation for the efficacy of the Semont-plus maneuver [56], reused under the Creative Commons Attribution 4.0 International License

A modification of the Semont maneuver is the so-called Semont Plus maneuver, which is based on the results of a mechanical model of the PPPV. During the first throw, the patient’s head (rotated 45° to the non-affected side) is thrown not only 90°, but at least 150 degrees to the affected side, whereby the otoconia moves much further in the desired direction (see Fig. 3, from their publication). In a tri-national study with 194 patients, the mean time to freedom from symptoms was significantly reduced from 3.6 to 1.8 days compared to the Semont maneuver [56]. In an analogous study, it was shown that the Semont Plus maneuver (mean time to freedom from symptoms 1.9 days) is also significantly superior to the Epley maneuver (mean time 3.3 days) [57]. It is important that the exercises are done three times in the morning, three times at noon and three times in the evening and that each position is maintained for 60 s. In addition, checks of the therapeutic effect after one week are indicated.

Horizontal canal PPPV Canalolithiais (hc-PPPV-Ca) and Cupulolithiasis (hc-PPPV-Cu). Various maneuvers have been developed for the treatment of hc-PPPV-Ca and hc-PPPV-Cu: For canalolithiasis (geotropic nystagmus), e.g., the modified roll maneuver, Gufoni maneuver, modified Zuma maneuver, and 12 h lying on the unaffected side For cupulolithiasis (apogeotropic nystagmus), head-shaking, modified Gufoni maneuver, otoconia reduction, or Zuma maneuvers. 3D simulations of six maneuvers for canalolithiasis show that only the modified roll maneuver with first 90° rotation to the affected side and the Zuma maneuver are theoretically effective for all forms. It is also important for the hc-PPPV that the maneuvers are carried out at least three times in a row, as a review shows [58].

### Menière’s disease

The current diagnostic criteria are described in Table 2 [59].

**Table 2:** Current diagnostic criteria for Menière’s disease [59]

Diagnostic criteria for Menière`s disease [59]:
Two or more episodes of vertigo, each lasting 20 min to 12 h
Audiometrically documented hearing loss in at least one examination < 2000 Hz > 30 dB and related to the episodes
Fluctuating tinnitus or aural fullness in the affected ear
Not better accounted for by another vestibular diagnosis

In laboratory testing, a typical finding in Menière’s disease is the discrepancy between pathological caloric excitability and a normal HIT [60] in contrast to vestibular migraine with mostly normal vestibular testing [61]; furthermore, hearing testing discriminates well between Menière’s disease and vestibular migraine [62].

There are a number of studies on the etiology of Menière’s disease that highlight the following aspects: 1) genetic causes, especially genes relevant to ional homeostasis in the inner ear, and 2) immunological mechanisms and association with immunological diseases (overview in [63]).

In the course of the disease, 13% of patients develop bilateral Menière’s disease after an average of 8 years (meta-analysis of nine studies, 1583 patients) [64], which should be considered when using ablative procedures.

### Therapy

Many studies continue to be published on the treatment of Menière’s disease. However, most of the therapy studies are not placebo-controlled, which is very problematic, especially due to the high placebo effect of 50% with regard to vertigo episodes [65].

Six Cochrane analyses from 2023 concluded that the evidence for the efficacy of the following measures is "low or very low": lower-salt diets, abstinence from coffee or alcohol, diuretics and betahistine at the doses studied so far (see below), intratympanic steroids (supported by a recent meta-analysis [66]), intratympanic gentamicin, surgical procedures, or methods based on pressure changes, such as the "Meniett device". In addition, a recent placebo-controlled study of intratympanic latanoprost (otherwise used to treat glaucoma) was negative [67].

So far, there is no evidence for the efficacy of betahistine at a dosage of up to 3 × 48 mg/d compared to placebo [68], so that higher dosages and/or combination with MAO-B inhibitors, such as selegiline or rasagiline, are suggested; the latter because betahistine is metabolized by 99% via MAO after oral administration. In a phase I study, it was shown that bioavailability increases by a factor of 100 with combination therapy [69]. As calculations show, with these high concentrations pharmacologically effective bindings to the histamine receptors can be achieved [69]. A placebo-controlled study with the combination therapy with strict inclusion criteria, clinically relevant endpoints, and long therapy and observation periods is planned. In addition to the direct increase of membrane permeability by activating the H1 receptors in the endolymphatic membrane, effects on immunomodulators that play a role in the pathogenesis of Menière’s disease have recently been reported as a mechanism of action [70], and proven [71].

## Vestibular migraine

### Clinical presentation and broadened clinical phenotype

Vestibular migraine (VM) is the most common cause of recurrent, spontaneous episodes of vertigo or dizziness. The diagnostic criteria for VM were first defined in 2012 by the Bárány Society and the International Headache Society and have remained unchanged since [72]. Recent work suggests that VM represents part of a broader spectrum of migraine-associated vestibular symptoms, many of which are clinically burdensome despite falling short of current diagnostic criteria. In a large 2025 cross-sectional study of 2801 newly diagnosed migraine patients, vestibular symptoms were present in 68.4%, whereas only 15.2% met formal criteria for VM, suggesting that clinically meaningful migraine-associated vestibular symptoms extend beyond currently classified VM [73]. This aligns with a recent review, which emphasizes the heterogeneity of the VM phenotype and suggests that VM should be understood as a broader clinical spectrum encompassing episodic, interictal, and chronic vestibular manifestations, visual motion sensitivity, and frequent overlap with functional vestibular disorders [74].

### Advancements in diagnostics

VM remains primarily a clinical diagnosis; however, there is increasing diagnostic innovation. A 2025 systematic review and meta-analysis including 14 studies in the review and 10 in the meta-analysis evaluated the effectiveness of machine learning algorithms in the diagnosis of VM. Artificial-intelligence (AI)-based models achieved encouraging diagnostic accuracy in pooled analyses (sensitivity of 0.85, specificity of 0.89, AUC of 0.94), yet current datasets remain too heterogeneous and lack external validation for routine clinical use [75]. A notable recent advancement in imaging is the use of delayed gadolinium-enhanced MRI of the endolymphatic space. A recent prospective case control study in 2025, including patients with VM, Menière’s disease, and healthy controls, showed that endolymphatic hydrops (EH) was detected in all three entities, indicating that its main diagnostic value is characterizing the overall imaging pattern rather than confirming/excluding its presence: EH may also occur in VM, but tends to be bilateral, low-grade and predominantly vestibular in distribution [76]. These findings support imaging as a complementary tool to supplement clinical criteria rather than replacing them.

### Acute and preventive treatment

Evidence for the acute treatment of VM is still very limited and remains unresolved as there are still no positive placebo-controlled studies. In the first adequately designed randomized controlled trial of acute treatment, rizatriptan failed to improve vertigo or dizziness at one hour versus placebo and showed only a limited 24-h benefit, arguing against routine use as an acute anti-vertigo therapy in VM [77]. Among preventive options, CGRP-pathway blockade has emerged as the most promising therapeutic advance. The INVESTMENT study was the first placebo-controlled trial evaluating the efficacy of a CGRP monoclonal antibody. Galcanezumab improved VM-specific handicap measures and reduced the number of dizzy days without reporting any serious adverse events [78]. A 2025 network meta-analysis concluded that Galcanezumab currently offers the best balance of efficacy, tolerability and evidence quality among evaluated preventive options, although larger comparative trials remain necessary [79].

### Differentiating vestibular migraine from Menière’s disease

VM and Menière’s disease have several clinical features in common and often present with overlapping symptoms due to shared pathophysiology including trigemino-vascular mechanisms and release of neuropeptides, such as CGRP [80]. Many patients meet diagnostic criteria for both conditions, complicating their differentiation in clinical practice. In a retrospective cohort of 119 patients fulfilling either VM or Menière’s disease criteria, 43 patients (36%) met criteria for both conditions, including 24 patients (20%) with both definite VM and definite Menière’s disease [81]. A study in 2025 including 188 patients with VM and 88 with Menière’s disease, sought to identify clinical features that allow to differentiate between VM and Menière’s disease: expectedly cochlear symptoms such as hearing problems and tinnitus, were more characteristic of Menière’s disease, whereas migrainous features including headache and photophobia were more frequent in VM. Importantly, no single symptom reliably distinguished the disorders. However, diagnostic accuracy improved when age at onset, cochlear symptoms, and migraine-associated features, among other variables, were considered together [61]. Objective vestibular testing may further aid the differential diagnosis: several studies have described a characteristic dissociation between preserved vestibular function on the vHIT and reduced caloric responses in Menière’s disease due to EH. In contrast, VM typically shows normal or non-specific vestibular test results [82]. Endolymphatic space imaging may provide supportive information as Menière’s disease more often showed pronounced and lateralized EH compared with VM, which was characterized by more often low-grade, bilateral and predominantly vestibular rather than cochlear endolymphatic space enlargement [76].

## Persisting vertigo, dizziness or gait imbalance

### Bilateral vestibulopathy

The current diagnostic criteria for bilateral vestibulopathy (BVP) are shown in Table 3 [83].

**Table 3:** Diagnostic criteria for bilateral vestibulopathy (BVP), modified [83]

Diagnostic criteria for bilateral vestibulopathy (BVP), modified [84]] A.Chronic vestibular syndrome with 1.Unsteadiness of stance and gait *and one or two* of the following symptoms: 2.Movement induced blurred vision or oscillopsia during walking or quick head/body movements 3.Worsening of postural imbalance or unsteadiness of gait in darkness and/or on uneven ground
B.No symptoms while sitting or lying down under static conditions
C.Bilaterally reduced or absent angular VOR function documented by bilaterally pathological horizontal angular VOR gain < 0.6, measured by the video-HIT/scleral-coil technique *and/o*r reduced caloric response (sum of bithermal max. peak SPV on each side < 6°/sec)
D.Not better accounted for by another disease

*BVP* bilateral vestibulopathy, *HIT* head impulse test, *SPV* slow-phase velocity, *VOR* vestibulo-ocular reflex

The term BVP is now well established internationally. As with AUVP, the role of vHIT as the first vestibular diagnostic procedure has been highlighted by a number of studies for BVP, which can be used to correctly classify most of the patients. If this is not pathological, caloric testing should be carried out. In addition to the well-known disruptions of spatial navigation (e.g., [84] with selective deficits in the combination of new paths), BVP leads in particular to impairments of executive functions and to a considerable impairment of the quality of life and high individual costs for the health system [85].

#### Etiology and genetics of BVP

Numerous studies are available on the etiology of BVP. In about 30–40%, the cause has so far remained unclear. In recent years, previous genetic studies have shown that two gene loci are particularly relevant for BVP: RFC1 and FGF14 (for an overview, see [86]). The mutations detected so far also depend on the respective comorbidities. The most common comorbidities of BVP are cerebellar ataxias and polyneuropathies, which are phenotypically referred to as "cerebellar ataxia, neuronopathy, vestibular areflexia syndrome (CANVAS)" when these three clinical signs are combined. The number of publications on this syndrome is steadily increasing, reaching at least 198 publications now (PubMed (CANVAS) AND (bilateral)). Three main aspects emerge, namely in relation to the phenotype and different underlying mutations and vice versa in relation to a particular genotype with different phenotypes. First, in two thirds of the cases, this is due to mutations in the "Replication Factor C1" (RFC1, "biallelic intronic AAGGG repeat expansion, 4p13; DNA polymerase accessory protein: synthesis and repair"). The severity of the disease correlates with the number of "repeats" [87]. Second, mutations in the first gene described can lead to other phenotypes, such as "ataxia with chronic cough" [88]—which is also of general medical importance—and can lead to MSA-C or a purely sensory neuropathy and only later move on to a full image of the CANVAS. Third, mutations in other genes, in particular "FGF14 GAA repeat expansions" can cause the same phenotype [89] or lead to a "late-onset cerebellar ataxia": SCA 27b [90].

From the "point of view" of the two gene loci, the following findings result:

"Replication Factor C1 (RFC1) GAA Repeat Expansions". Biallelic RFC1 expansions—which also play an important role in downbeat nystagmus (see also below)—were detected in 10/127 patients (8%) with isolated BVP, making them the most common monogenic cause of BVP [91].

Fibroblast growth factor 14 (FGF14). This locus was genotyped in 45 patients with the combination of cerebellar ataxia without and/or with polyneuropathy and/or with BVP (these were negative for the above-mentioned biallelic "RFC1 expansions"). The incidence of FGF14 expansions was 38% (17/45) in the overall cohort, 38% (5/13) in the cerebellar ataxia plus polyneuropathy subgroup, 43% (9/21) in the cerebellar ataxia plus BVP subgroup, and 27% (3/11) in patients with all three diagnoses. Overall, BVP was found in 75% (12/16) of GAA-FGF14-positive patients [89]. However, FGF14 GAA expansions are overall a rare cause of primary BVP syndromes [92].

Finally, a genome-wide association study (GWAS) in a European cohort of 132 individuals with “idiopathic BVP” and 3,410 unaffected controls identified the ZNF91/LINC01224 locus at chromosome 19 as a major genetic risk factor for BVP, implicating transcriptional regulatory mechanisms in disease susceptibility, thereby predicting a functional link between transcriptional regulation, retrotransposon silencing, and vestibular pathology, with age-related decline in ZNF91 expression potentially amplifying genetic susceptibility [93]

#### Video-head-impulse testing (vHIT) in RFC1 and SCA 27b

A link between the intronic RFC1 repeat expansion size and the severity of pursuit eye movement abnormalities, deterioration in visually enhanced VOR, and vHIT-gains was observed in one study [94]. The potential value of vHIT as a biomarker for monitoring progression of CANVAS/RFC1-related ataxia was summarized in a recent meta-analysis of individual patient vHIT-data [86]. In a dataset containing 146 patients, severe average (± 1 standard deviation) vHIT-gain reductions in both horizontal and vertical canals (ranging from 0.29 ± 0.03 to 0.39 ± 0.03) were found. Furthermore, a significant association between reduced horizontal vHIT-gains and disease duration was identified, supporting the notion that intra-individual decline in VOR-gains reflects the underlying neurodegenerative disease process.

Importantly, in contrast to RFC1-related ataxia, BVP seems much less frequent in SCA 27b (seen in only 10% of SCA 27b patients with FGF14 (GAA) ≥ 250 in one study [95]) and if present, gain reductions seen on horizontal vHIT were moderate only (averaging at 0.44, range = 0.3–0.53). Another study indicated that vHIT-gain reductions in SCA 27b were most pronounced in the posterior canals (average = 0.44), followed by anterior (0.54) and horizontal canals (0.83) [96].

#### Distinguishing RFC1-related ataxia from SCA 27b

**Fig. 4:**
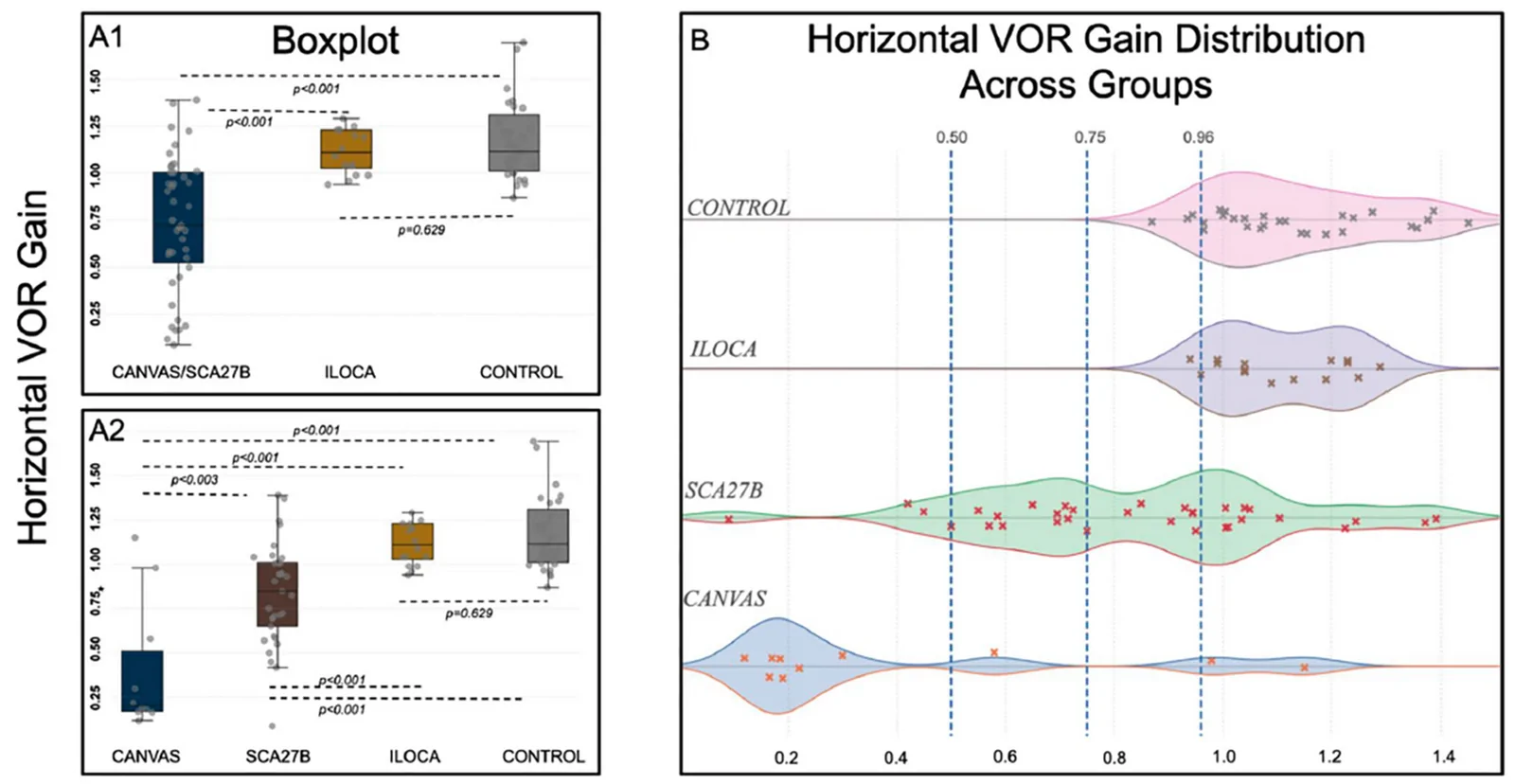
Horizontal vHIT-derived vestibulo-ocular reflex (VOR) gain across groups. A1) Boxplots comparing combined CANVAS/SCA27B versus ILOCA and controls, showing significantly lower VOR gains in the genetic groups (*p* < 0.001). A2) Separate boxplots for CANVAS, SCA 27b, ILOCA, and controls demonstrate progressively reduced gains, lowest in CANVAS. Kruskal–Wallis test p < 0.001. B) Density plots of horizontal VOR gain distribution across predefined intervals highlight clustering of low gains in CANVAS, intermediate in SCA 27b, and near normal in ILOCA and controls. Dashed vertical lines indicate clinically established cutoff values for VOR gain (0.50, 0.75, 0.96). *IQR* interquartile range, *VOR* vestibulo-ocular reflex, *vHIT* video head impulse test [98]. Reproduced with permission from Springer Nature

While the phenotype of RFC1-related ataxia and SCA 27b may overlap, quantitative VOR-testing may facilitate its distinction. Specifically, one study comparing vHIT-gains of the horizontal canals demonstrated more severe VOR-gain reductions in RFC1-related ataxia compared to SCA 27b (0.19 ± 0.13 vs. 0.52 ± 0.14) [97]. In another retrospective, comparative study, the prevalence of BVP (defined as VOR-gain < 0.6 on vHIT) varied significantly between RFC1-related ataxia (80%), SCA 27b (18%) and idiopathic late-onset cerebellar ataxia (ILOCA, 0%) [98]. Confirming previous work, average horizontal VOR-gains were significantly lower in RFC1-related ataxia (0.22) and SCA 27b (0.85) than in ILOCA (1.11) (see Fig. 4, from their publication). A cut-off VOR-gain value of 0.50 was identified to distinguish RFC1-related ataxia from SCA 27b reliably (91% specificity and 70% sensitivity) in this cohort.

#### Therapy

Treatment of choice in BVP is lifelong daily balance training. So far, however, there is a lack of methodologically high-quality studies to determine effective training parameters. Compared to AUVP, a larger amount of training is necessary. There are variations both for practicing the combination with head movements (from three to five times a day for a total duration of 20 to 40 min) and for practicing balance and gait exercises (for at least 20 min daily) [18]. Another effective measure has proven to be "noisy galvanic vestibular stimulation" [99], which is ideally combined with lifelong daily vestibular rehabilitation. Finally, the vestibular implant is appearing on the horizon [100].

Responses to 4-aminopyridine and acetyl-DL-leucine in RFC1-related disorders are rare and limited, suggesting that neurodegenerative dysfunction of cerebellar circuits in RFC1-disease—in contrast to certain genetic cerebellar disorders, such as SCA 27b—is unlikely to be improved to clinically relevant thresholds by these agents [101].

#### Cerebellar dizziness

Cerebellar dizziness can manifest itself as 1) permanent dizziness with unsteadiness of gait, as in neurodegenerative, genetic, or autoimmune cerebellar diseases; 2) acute central syndrome in a cerebellar infarction (often acute imbalance syndrome); 3) recurrent episodes, such as episodic ataxia type 2; and 4) as a combination of these symptoms (e.g., [102]). A key to the diagnostic classification is a careful examination of the ocular motor function with the question of cerebellar ocular motor disorders, such as DBN, saccadic smooth pursuit, or gaze-evoked nystagmus.

#### Downbeat nystagmus

Downbeat nystagmus (DBN) is the most common form of acquired persistent nystagmus. The main symptoms are imbalance and oscillopsia with intensification in the side view. In 30% to 50% of cases, the etiology remained unclear. However, it is becoming increasingly clear that most of these "idiopathic" cases have a genetic cause, which also has consequences for treatment (especially "personalized medicine"). In one study, 170 patients were examined for the presence of "FGF-14 GAA repeat expansions" (cause of SCA 27b, [90]). Furthermore, the response to treatment with 4-aminopyridine was analyzed as a function of the presence of these genetic changes [95]. In 48% (82/170) of the entire cohort with "idiopathic DBN" there were "FGF-14 GAA repeat expansions", in 61% (42/69) with DBN plus cerebellar oculomotor disorders (DBN syndrome) and in 72% (23/32) with DBN plus cerebellar ataxia. Likewise, DBN was identified in 50% of cases both in a French-Canadian cohort (with ≥ 200 repeats in the FGF14-gene, [103]) and in a European study (with ≥ 250 repeats in the FGF14-gene, [104]). In two US case series, DBN was found in 70% (48/69) [105]) and in 85% (17/20) of cases [96], respectively. In comparison to other spinocerebellar ataxias (SCA1/2/3/6/8), spontaneous and positional DBN was significantly more frequent in SCA 27b in one retrospective case series (*n* = 20), whereas gaze-evoked nystagmus, rebound nystagmus, and impaired pursuit eye movements occurred at similar rates [96]. Furthermore, in one cohort 63% of patients with ataxia and DBN of unknown origin were linked to FGF14 repeat expansion [106].

Patients with FGF-14 GAA repeat expansions showed an 80% response rate to treatment compared to 31% of patients without it in one study [95], with a positive predictive value in response to this therapy for a mutation being 95%. These findings are in line with those from several other studies that reported a positive (sometimes marked) subjective (clinician or self-reported) treatment response to (sustained-release) 4-aminopyridine in 71 to 86% of SCA 27b patients [105–107]. Likewise, placebo-controlled VOG-data, available from four patients carrying an FGF14 (GAA) ≥ 250 expansion, showed a significant decrease in slow-phase DBN velocity with 4-aminopyridine, but not placebo in one study [95]. This closes the circle between etiology, pathophysiology, and therapy with 4-aminopyridine in DBN. Like BVP, RCF-1 (CANVAS gene) also plays a role. Biallelic mutations in this gene can also manifest as DBN: they were found in 15 out of 65 (23%) with "idiopathic" DBN (without "FGF-14 GAA repeat expansions") [108]. In the latter cohort, the response to 4-aminopyridine was less than 5%, which can be explained pathophysiologically, as this mutation leads to neurodegeneration, while "FGF-14 GAA repeat expansions" result in a reduction in the excitability of Purkinje cells and 4-aminopyridine increases it. Finally, more and more patients with overlap syndrome are described with RFC-1 and FGF-14 mutations [109].

#### Episodic ataxia type 2

Episodic ataxia type 2 (EA2) is characterized by recurrent episodes of ataxia and dizziness that usually last for hours, which can be triggered by physical activity, stress, or alcohol, but can also occur spontaneously. In the interval, central ocular motor disorders are found in > 90% of patients, in particular a clear DBN, which is an important differential diagnostic criterion also to distinguish it from VM. EA2 is one of the so-called CACNA1A neurodevelopmental diseases. A placebo-controlled "double cross-over" study showed that both 4-aminopyridine (fampridine, 2 × 10 mg/d) and acetazolamide (750 mg/d) are effective for prophylaxis [110]. However, acetazolamide had significantly more adverse effects, so that after the study, 24 patients took 4-aminopyridine and only 3 took acetazolamide. The positive effect of 4-aminopyridine in EA2 is supported by a recent case series [111].

Another molecule used to treat cerebellar ataxia, lysosomal diseases and pre-stages of Parkinson’s disease is the modified amino acid N-acetyl-L-leucine (NALL). Positive effects on lysosomal diseases have been demonstrated in animal models as well as a phase 3 study in Niemann-Pick type C (NPC [112]), a phase 3 study in ataxia telangiectasia [113], gangliosidosis type 2 (GM2, phase 2 study), and "CACNA1A neurodevelopmental disease" [114]. The same drug also had an obvious neuroprotective effect in 2 patients with pre-stages of Parkinson’s disease [115]. Finally, a neuroprotective effect of NALL was also shown in a long-term study in patients with NPC, corresponding to a 118% reduction in annual disease progression [116].

#### Functional dizziness

Functional dizziness (FD)—the umbrella term for different subtypes, including persistent postural-perceptual dizziness (PPPD) and phobic postural dizziness—is increasingly recognized as the most common chronic functional vestibular disorder. It may follow acute or episodic vestibular disorders including AUVP and VM [117, 118]. Prospective studies of patients followed for 3–12 months after acute or episodic vestibular disorders suggest that FD develops in approximately one out of four (25%) patients [119], while a cohort study of episodic vestibular disorders reported PPPD in 24 of 376 patients (6.4%) and identified VM as a precipitating condition in 16.5%, Menière’s disease in 3.9%, and peripheral paroxysmal positional vertigo in 2.3% [120]. Pathophysiology encompasses a maladaptive multisensory integration and a high vigilance for body sensation. A review conducted in 2025 highlighted alterations in brain structure, activity, and connectivity [121]. Experimental work has demonstrated altered visual behavior with gaze directed predominantly downward towards the ground, frequent large amplitude vertical gaze shifts and shorter fixation durations, a pattern possibly reflecting an anxiety driven search for potential postural support [122]. A recent cross-sectional study showed that psychological factors, particularly illness-related cognitive appraisals, are associated with PPPD symptom dimensions, including balance vigilance, and may mediate their relationship with FD diagnosis [123]. Together these findings support a complex maladaptive network-model in which altered sensory weighting, dysfunctional postural control mechanisms and psychological factors contribute to the perpetuation of chronic dizziness.

#### Current gaps and outlook

While recent publications have focused on improving the diagnostic accuracy for distinguishing stroke from AUVP at the bedside, identifying acute episodes of VM in the ED is still challenging. Often, other (non-structural) central causes of acute/vertigo are excluded from study populations, or they do not meet diagnostic criteria yet for VM (presenting with their first or second episode). Thus, adapting bedside algorithms to the clinical presentation of acute VM episodes may be one of the more urgent tasks.

Furthermore, knowledge about how to best treat posterior-circulation stroke presenting as AVS in the hyperacute phase is still very limited. This is especially true if NIHSS is low or zero and obvious focal neurologic signs are missing. A recent systematic review [124] and a retrospective single-center trial [125] emphasized the lack of data to drive any firm recommendation about treating otherwise eligible patients presenting with AVS with intravenous thrombolysis (IVT) and endovascular thrombectomy (EVT) and concluded that judgments must be made on a case-by-case basis. Likewise, the risk of over-referral and over-treatment must be considered in AVS patients, taking into account that improvement of outcome by use of IVT/EVT has not been demonstrated.

In terms of treatment, there is still a very high need for controlled clinical trials, comparing the different treatment maneuvers for PPPV, treatment of AUVP, Menière’s disease, VM, vestibular paroxysmia, and FD with validated and clinical meaningful endpoints.

## Conclusions

Major progress has been made in terms of the pathophysiology, etiology, genetics, and diagnostic criteria of peripheral, central, and functional vestibular diseases as well as ocular motor disorders. However, this knowledge must be transferred into clinical practice, which is still challenging and requires systematic teaching of doctors to increase knowledge and improve skills in applying the different tests in clinical practice on a high level. In terms of treatment, as stated above, there is still a very great need for state-of-the art clinical trials applying approved therapies.

## Data Availability

No new data were created or analyzed in this study. Data sharing is not applicable to this article.
